# Clinical Profile of Giant Cell Arteritis and Polymyalgia Rheumatica in a Tertiary Care Hospital in South India

**DOI:** 10.7759/cureus.104390

**Published:** 2026-02-27

**Authors:** Abraham George N, Manikandan Gopal, John Mathew, Ruchika Goel, Meera Thomas

**Affiliations:** 1 Department of Clinical Immunology and Rheumatology, Christian Medical College Vellore, Vellore, IND; 2 Department of General Pathology, Christian Medical College Vellore, Vellore, IND

**Keywords:** aortoarteritis, india, large vessel vasculitis, temporal arteritis, temporal artery biopsy, temporal artery doppler

## Abstract

Introduction

This study examines the clinical profiles of patients with giant cell arteritis (GCA) and polymyalgia rheumatica (PMR) attending a tertiary care center in South India.

Methods

Electronic medical records of patients diagnosed with GCA and PMR from 2005 to 2021 were reviewed.

Results

There were 10 patients with GCA. The mean age of the patients was 64.9 (±8) years. There were six (60%) male patients and four (40%) female patients. Headache was seen in eight (80%) patients. Four (40%) had visual symptoms. Three (30%) had jaw claudication and temporal artery tenderness each. Only one (10%) patient with GCA had PMR. Temporal artery Doppler was performed in nine out of 10 cases, of which four (44.4%) were abnormal. A temporal artery biopsy was performed in five cases, of which three (60%) were diagnostic. The mean erythrocyte sedimentation rate (ESR) was 85.2 (±29.5) mm, and the mean C-reactive protein (CRP) was 38.12 (±50.8) mg/dL. Treatment and follow-up were noted.

There were five patients with PMR. The mean age of the patients was 55.8 (±1.5) years. There were two (40%) men and three (60%) women. The mean ESR was 60 (±31.9) mm/hour. The mean CRP was 65 (±66.8) mg/L.

Conclusions

GCA and PMR are uncommon in this population. Patients with GCA in the region are younger, and the disease appears to be more common in men. The proportion of patients with PMR symptoms and those with a positive temporal artery biopsy was less common in our population. Most patients with GCA and PMR respond well to steroids and other immunosuppressants.

## Introduction

Giant cell arteritis (GCA) is a large vessel vasculitis that typically affects patients over 50 years of age [1]. The disease is most common in Scandinavian countries, followed by North America. However, the incidence is lower in Southern European countries and Mediterranean countries [2,3]. The disease is sporadic in Asia and India [3-5]. Polymyalgia rheumatica (PMR) is a syndrome of pain and stiffness in the shoulder, hip, and neck associated with raised inflammatory markers, which is also seen in patients older than 50 years. GCA and PMR share a similar genetic background and epidemiology, and the two conditions often co-occur [6-8]. Similar to GCA, PMR is also uncommon in India [5,9]. Data are scarce regarding the presentation, diagnostic features, treatment response, and prognosis of patients with GCA and PMR in the region and the country. This study tries to explore the clinical profile of GCA and PMR in our population, which includes clinical features, various blood laboratory parameters, and diagnostic investigations such as temporal artery Doppler and temporal artery biopsy, observe if it confirms the findings in the few available previous studies from the region, and observe the differences from the profile seen in other parts of the world. The study also examines the treatment given and the response to it.

## Materials and methods

Our electronic database was searched for "giant cell arteritis," "GCA," "temporal arteritis," "polymyalgia rheumatica," and "PMR," and the medical reports of out-patients and the discharge summaries of in-patients with above diagnosis among the patients under the care of the Department of Clinical Immunology and Rheumatology over a period of 16 years (2005-2021) were retrieved. The principal investigator reviewed each of these records. Duplications were removed. Charts of 12 patients with GCA and 12 patients with PMR mentioned in the diagnosis were reviewed. All patients who fulfilled the ACR 1990 criteria (Table 1) [10] for GCA and Chuang's criteria (Table 2) [11] for PMR were included in the study. Chuang's criteria were selected for PMR because the details were more easily available on the records.

**Table 1:** ACR 1990 criteria for the classification of giant cell arteritis For purposes of classification, a patient shall be said to have giant cell (temporal) arteritis if at least three of these five criteria are present. The presence of any three or more criteria yields a sensitivity of 93.5% and a specificity of 91.2%. ACR: American College of Rheumatology, ESR: erythrocyte sedimentation rate Reproduced with permission from John Wiley and Sons Source: [10]

Criterion	Definition
Age at disease onset ≥ 50 years	Development of symptoms or findings beginning at age 50 or older
New headache	New onset of a new type of localized pain in the head
Temporal artery abnormality	Temporal artery tenderness to palpation or decreased pulsation, unrelated to arteriosclerosis of cervical arteries
Elevated ESR	ESR ≥ 50 mm/hour by the Westergren method
Abnormal temporal artery biopsy	Biopsy specimen with artery showing vasculitis characterized by a predominance of mononuclear cell infiltration or granulomatous inflammation, usually with multinucleated giant cells

**Table 2:** Chuang et al. criteria for PMR ESR: erythrocyte sedimentation rate, PMR: polymyalgia rheumatica Source: [11]

Criteria
Age at onset ≥ 50 years
B/l moderate to severe aching and stiffness persisting for ≥1 month involving two of the following areas: neck or torso, shoulders or proximal region of the arms, and hips of proximal aspects of the thigh
ESR ≥ 40 mm/hour
Exclusion of all other diagnoses of mimicking entities
All criteria are required for classification

Those who were detected to have other rheumatological diseases were excluded. Clinical features, laboratory investigations, temporal artery Doppler, and biopsy findings were noted. Follow-up was noted where available. Data was entered into an Excel sheet (Microsoft Corp., Redmond, WA) and analyzed. Mean values were calculated for approximately normally distributed data, and the median was calculated for skewed data.

## Results

Giant cell arteritis

Out of the 12 patients suspected to have GCA, 10 patients in whom other diseases were excluded and who fulfilled the ACR 1990 criteria for GCA were included in the study. The mean age of the patients was 64.9 (±8) years. There were six (60%) male patients and four (40%) female patients (male/female ratio: 1.5:1). Headache and weight loss were the most common symptoms, followed by fever and visual impairment. Other clinical features are mentioned in Table 3.

**Table 3:** Clinical features of patients with GCA and PMR GCA: giant cell arteritis, PMR: polymyalgia rheumatica

Feature	Number of patients (%)
GCA	10
Headache	8 (80%)
Weight loss	8 (80%)
Fever	6 (60%)
Visual impairment	4 (40%)
Jaw claudication	3 (30%)
Temporal artery tenderness	3 (30%)
Arthralgia	1 (10%)
Polymyalgia rheumatica	1 (10%)
Diplopia	1 (10%)
Co-existing PMR	1 (10%)
PMR	5
Fever	2 (40%)
Arthralgia (peripheral)	2 (40%)
Weight loss	1 (20%)
Co-existing GCA	1 (20%)

Temporal artery Doppler was done in nine out of the 10 cases, of which four (44.4%) were abnormal (Table 4).

**Table 4:** Temporal artery Doppler findings in 4/9 patients in which it was abnormal

Observations	Number of patients (%)
Thickening, nodularity, luminal narrowing	1 (20%)
Reduced caliber	1 (20%)
Diffuse thickening	1 (20%)
Halo sign	1 (20%)

**Figure 1:**
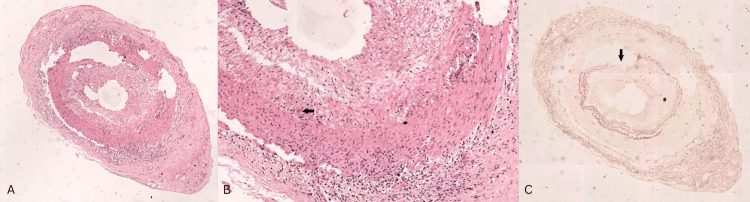
Temporal artery biopsy of one patient with giant cell arteritis A: H&E (40×) staining showing transmural infiltration of inflammatory cells. B: Higher magnification (H&E, 200×): giant cells (arrow). C: Orcein staining showing a break in the internal elastic lamina (arrow). H&E: hematoxylin and eosin

Temporal artery biopsy was performed in five cases, of which three (60%) exhibited vasculitis features with giant cells, with or without granuloma (the biopsy of one patient is shown in Figure 1A-1C). Of the other two biopsies, one showed focal intramural chronic inflammation, and the other showed mild intimal fibrosis, focal fragmentation, or loss of the internal elastic lamina.

The mean ESR was 85.2 (±29.5) mm, and the mean CRP was 38.12 (±50.8) mg/L. Other laboratory parameters are given in Table 5.

**Table 5:** Mean laboratory values for patients with GCA and PMR Hb: hemoglobin, TC: total white blood cell count, Cr: serum creatinine, Alb: serum albumin, SD: standard deviation, ESR: erythrocyte sedimentation rate, CRP: C-reactive protein, GCA: giant cell arteritis, PMR: polymyalgia rheumatica

Mean laboratory values at presentation								Mean values at last follow-up	
Disease	Hb (g/dL) (SD)	TC (/mm3) (SD)	Platelet (Lakhs/mm3) (SD)	Cr (mg/dL) (SD)	Alb (g/dL) (SD)	ESR (mm) (SD)	CRP (mg/dL) (SD)	ESR (mm) (SD)	CRP (mg/L) (SD)
Normal range	Males: 13-17, females: 11-15	4,000-12,000	1.5-4.5	0.5-1.4	3.5-5	Males: up to 20, females: up to 30	<6	Males: up to 20, females: up to 30	<6
GCA	11.7 (1.8)	10,050 (1,731)	3.08 (1.2)	0.78 (0.2)	3.56 (0.6)	85.2 (28)	38 (47.9)	16.8 (6.9)	6.7(2.7)
PMR	11.1 (1.91)	10,540 (1,755)	3.35 (0.86)	0.78 (0.18)	3.33 (0.90)	60 (31.9)	65 (66.8)	3.9 (2)	1.08 (0.54)

The median follow-up was 55 months. All patients were treated with steroids. Most patients were initiated on 1 mg/kg prednisolone equivalent steroids. However, steroid doses varied as some of them were already on treatment when presented to our center. All patients (except two who did not review after the first visit) were started on steroid-sparing agents: azathioprine (4), methotrexate (1), mycophenolate (2), and tocilizumab, along with methotrexate (1). Tocilizumab, along with methotrexate, was started upfront for one patient because of glaucoma, which necessitated a lower dose of steroids. Among the 10 patients of GCA, only five patients had a follow-up of more than one year. The median follow-up for these five patients was 90 months. One of those five patients, who was on azathioprine, developed new-onset optic neuropathy while on treatment. She also had azathioprine-induced leucopenia. She was given intravenous immunoglobulin (IVIG) 2 g/kg and 0.5 mg/kg prednisolone equivalent steroids (higher dose steroids were avoided in view of severe osteoporosis). Also, the immunosuppressant was changed to mycophenolate. Thereafter, her disease stabilized and was doing well on low-dose steroids (and mycophenolate) during the available nine years of follow-up. Of the four other patients whose follow-up was available, one patient who was on mycophenolate for two years had persistent headaches and one episode of diplopia, and finally had visual impairment on treatment and was shifted to tocilizumab. The three others were stable. Steroids were tapered and stopped for one patient (on azathioprine), and the other two were doing well on low-dose steroids (one on methotrexate and the other on azathioprine).

Polymyalgia rheumatica

Out of 12 patients suspected to have PMR, five patients whose other diseases were excluded and who fulfilled the Chuang criteria were included in the study. The mean age of the patients was 55.8 (±1.48) years. There were two (40%) male patients and three (60%) female patients (male/female ratio: 1:1.5). Out of the five patients, two had fever (40%), two had arthralgia (40%), one had weight loss (20%), and one had co-existing GCA (20%). The mean ESR was 60 (±31.9) mm/hour. The mean CRP was 65 (±66.8) mg/L. Other laboratory values are shown in Table 3. All patients were started on steroids (10-20 mg of prednisolone equivalent; the patient with GCA was initiated on 60 mg prednisolone). All patients were started on steroid-sparing agents. Four of them were started on methotrexate, and the other patient (who had co-existing GCA) was started on azathioprine. Among these patients, one patient did not have further follow-up. The other four patients had a median follow-up of 71 months. The patient with co-existent GCA (who was started on azathioprine) had developed optic neuropathy while on treatment and was given IVIG and 0.5 mg/kg prednisolone equivalent steroids, and was changed to mycophenolate. Thereafter, her disease was stable during the available nine years of follow-up and was on low-dose steroids during the last visit. Other patients were stable during the available follow-up. Steroids were tapered and stopped for one patient, and the three others were doing well on low-dose steroids (all four of them on methotrexate).

## Discussion

Giant cell arteritis is a large vessel vasculitis that primarily affects older individuals. The study is limited by a small sample size, although our center is a tertiary care institute with a large number of patients with rheumatology. However, it has to be noted that the number is comparable to other studies from India [5,12,13]. Hence, the low sample size might also reflect the low incidence of GCA in this population. Underdiagnosis is another reason that might have contributed to this. Additionally, although less likely, some patients may have been treated in other departments within our center. In our study, the mean age of onset of the disease was 64.9±8 years, comparable to other studies from India. The male/female ratio in this study was 1.5:1. A male-predominant trend is also observed in other studies in the region [5,12] and is in contrast to that seen in other regions across the world [14-18]. The proportion of patients with headache, temporal artery tenderness, and polymyalgia rheumatica was somewhat lower than in other studies. Other findings were similar, although variations were observed among different studies [5,12,13]. Temporal artery Doppler and biopsy findings are discussed below. PMR symptoms were less common in patients with GCA than in other studies. Overall, the findings were in alignment with the previous studies from the region. Table 6 shows a comparison of the present study with earlier studies from India.

**Table 6:** Comparison of findings of the present study with previous Indian studies TA: temporal artery, M: male, F: female, PMR: polymyalgia rheumatica

Variable	Present study (n=10)	Sharma et al. (2015)(n=17) [5]	Mathew et al. (2012) (n=15) [12]	Singh et al. (2010)(n=16) [13]
Mean age (years)	64.9	67	67.5	66.5
M/F	1.5:1	2.4:1	1.5:1	1:1
Headache (%)	80	100	100	93.7
Visual symptoms (%)	40	58.8	20	18.8
Jaw claudication (%)	30	53	20	56.2
TA tenderness (%)	30	70.6	100	68.7
TA Doppler (%)	44.4	66.6	-	37.5
TA biopsy diagnostic (%)	60	38.5	69	91
PMR	10	41	53.3	31.3

The findings were also similar to studies from other parts of the world, with some important differences. The patient numbers are much higher in studies from other parts of the world. The mean age of the patients was slightly lower than that in other studies. As mentioned above, the disease trended to be more common in men in our region, which is in contrast to what is seen in other parts of the world, where the disease appeared to be more common in women. PMR symptoms were more common in patients with GCA in other countries. Temporal artery Doppler and biopsy findings are discussed below. Table 7 shows a comparison of the present study with studies from other parts of the world.

**Table 7:** Comparison of findings of the present study with previous studies worldwide TA: temporal artery, M: male, F: female, PMR: polymyalgia rheumatica

Variable	Present study (n=10)	Fernández-Lozano et al. (2024), Spain (n=1,675) [14]	Garvey et al. (2021), USA (n=304) [15]	Lyne et al. (2022), New Zealand (n=142) [16]	Sun et al. (2016), China (n=70) [17]	Yamaguchi et al. (2023), Japan (n=36) [18]
Mean age (years)	64.9	76.9	77.5	66.5	66.4	76
M/F	1.5:1	1:2.3	1:3.3	1:1.9	1:1	1:1.25
Headache (%)	80	79.9	73	79.7	68.6	78
Visual symptoms (%)	40	36.1	18	48	27.1	28
Jaw claudication (%)	30	35.7	45	44	30	39
TA tenderness (%)	30	49.2	31	55	30	-
TA Doppler (%)	44.4	67.7	-	-	-	-
TA biopsy diagnostic (%)	60	62.8	86	74	71.4	82
PMR	10	41.8	29	54.4	27.1	44

The sample size is too low to draw meaningful conclusions regarding the sensitivity, specificity, or predictive values of diagnostic investigations such as temporal artery Doppler and biopsy. Temporal artery Doppler was abnormal in four of nine cases. Only a few studies had commented on the proportion of patients who had positive temporal artery Doppler, and the same was slightly higher than in the present study. Since it is negative in a significant number of patients, the same may not be sufficiently sensitive to rule out the diagnosis. Temporal artery biopsy was diagnostic in 60% of cases in the present study. Similar to Doppler, although it can assist in diagnosis, temporal artery biopsy may not be able to rule out the diagnosis well enough. The well-documented "skip lesion" phenomenon might contribute to this. This percentage was higher in most other studies from the region, as well as in those from other parts of the world [5,12-18].

There were no studies from India on the profile of patients with polymyalgia rheumatica. The low number of patients in the study reflects the low incidence of the disease in our country, although underdiagnosis may have contributed to this, as seen in GCA.

Limitations

This is a retrospective study from a single center. The sample size of the study is small. This is mainly because the disease is sporadic in the population, and the sample size is comparable to that of other studies from the region. There might be referral bias in the study owing to the tertiary nature of our center. Also, Chuang's criteria are used to classify patients as having PMR rather than the more recent ACR/EULAR criteria. If the latter criterion were used, all the current patients are expected to be included, and one or two more patients might also have been included. This may be significant considering the very low number of patients with PMR. Also, the ACR 1990 criteria were used for classifying GCA, as it was the standard criteria at the time of the study. There was a single reviewer with no cross verification of data, and there is a lack of a standardized data extraction process. The study does not mention details of vascular imaging or PET in GCA cases, which is being increasingly used nowadays, but was not widely used in earlier days. Because our center is a tertiary care center, follow-up data are not available for many patients, as many were lost to follow-up, which makes it unable to make reliable conclusions on treatment response.

## Conclusions

GCA and PMR are uncommon in this population. Headache, weight loss, fever, and visual impairment were the most common symptoms of GCA. Temporal artery Doppler and biopsy can assist in diagnosis, but cannot reliably rule out the diagnosis. However, the sample size is too low to draw meaningful conclusions. Patients with GCA in the region are younger, and the disease appears to be more common in men. PMR symptoms may be less common than previously thought. The proportion of patients with positive temporal artery biopsy was lower in our population. Larger multicenter studies are required to confirm the trends observed in the above study. Most patients with GCA and PMR respond well to steroids and other immunosuppressants.
